# Wild bees and their nests host *Paenibacillus* bacteria with functional potential of avail

**DOI:** 10.1186/s40168-018-0614-1

**Published:** 2018-12-22

**Authors:** Alexander Keller, Annette Brandel, Mira C. Becker, Rebecca Balles, Usama Ramadan Abdelmohsen, Markus J. Ankenbrand, Wiebke Sickel

**Affiliations:** 1 Department of Animal Ecology and Tropical Biology, Biocenter, University of Würzburg, Am Hubland, 97074 Würzburg, Germany; 2 Present Address: Center for Computational and Theoretical Biology, Biocenter, University of Würzburg, Hubland Nord, 97074 Würzburg, Germany; 3 Present Address: Department of Bioinformatics, Biocenter, University of Würzburg, Am Hubland, 97074 Würzburg, Germany; 4 Present Address: Faculty of Biology, Albert-Ludwigs-University Freiburg, Schänzlestraße 1, 79104 Freiburg, Germany; 5 Present Address: BIOSS Centre for Biological Signalling Studies, Albert-Ludwigs-University Freiburg, Schänzlestraße 18, 79104 Freiburg, Germany; 6 Present Address: Department of Behavioral Physiology & Sociobiology, Biocenter, University of Würzburg, Am Hubland, 97074 Würzburg, Germany; 7 Department of Botany II, Julius-von-Sachs Institute for Biological Sciences, University of Würzburg, Julius-von-Sachs-Platz 3, 97082 Würzburg, Germany; 8 Present Address: Department of Pharmacognosy, Faculty of Pharmacy, Minia University, Minia, 61519 Egypt; 9 Present Address: Molecular Biology of the Rhizosphere, Institute of Crop Science and Resource Conservation, Nussallee 13, 53115 Bonn, Germany

**Keywords:** 16S metabarcoding, American foulbrood, Anti-microbial activity, Bacterial genomics, Bioassays, European foulbrood, Paenibacterin, Phylogenomics, Bee disease, Pathogen vector

## Abstract

**Background:**

In previous studies, the gram-positive firmicute genus *Paenibacillus* was found with significant abundances in nests of wild solitary bees. *Paenibacillus larvae* is well-known for beekeepers as a severe pathogen causing the fatal honey bee disease American foulbrood, and other members of the genus are either secondary invaders of European foulbrood or considered a threat to honey bees. We thus investigated whether *Paenibacillus* is a common bacterium associated with various wild bees and hence poses a latent threat to honey bees visiting the same flowers.

**Results:**

We collected 202 samples from 82 individuals or nests of 13 bee species at the same location and screened each for *Paenibacillus* using high-throughput sequencing-based 16S metabarcoding. We then isolated the identified strain *Paenibacillus* MBD-MB06 from a solitary bee nest and sequenced its genome. We did find conserved toxin genes and such encoding for chitin-binding proteins, yet none specifically related to foulbrood virulence or chitinases. Phylogenomic analysis revealed a closer relationship to strains of root-associated *Paenibacillus* rather than strains causing foulbrood or other accompanying diseases. We found anti-microbial evidence within the genome, confirmed by experimental bioassays with strong growth inhibition of selected fungi as well as gram-positive and gram-negative bacteria.

**Conclusions:**

The isolated wild bee associate *Paenibacillus* MBD-MB06 is a common, but irregularly occurring part of wild bee microbiomes, present on adult body surfaces and guts and within nests especially in megachilids. It was phylogenetically and functionally distinct from harmful members causing honey bee colony diseases, although it shared few conserved proteins putatively toxic to insects that might indicate ancestral predisposition for the evolution of insect pathogens within the group. By contrast, our strain showed anti-microbial capabilities and the genome further indicates abilities for chitin-binding and biofilm-forming, suggesting it is likely a useful associate to avoid fungal penetration of the bee cuticula and a beneficial inhabitant of nests to repress fungal threats in humid and nutrient-rich environments of wild bee nests.

**Electronic supplementary material:**

The online version of this article (10.1186/s40168-018-0614-1) contains supplementary material, which is available to authorized users.

## Introduction

In insects, and particularly honey bees, the genus *Paenibacillus* includes severe pathogens such as the causative agent of the fatal honey bee disease American foulbrood, *Paenibacillus larvae* [1]. Infections can result in severe colony losses, and it is one of the most widespread as well as destructive bee brood diseases [1]. *Paenibacillus alvei* is known to accompany European foulbrood diseases as a secondary invader [2, 3], and *Paenibacillus apiarius* is also considered to be a threat to honey bees, although it has not received much attention so far [3, 4].

Recently, *Paenibacillus* bacteria were reported from nests of solitary bee species, *Osmia bicornis* [5, 6] and *Osmia cornuta* [7], but the larval specimen investigated seemed properly developed and without signs of unhealthy conditions [5]. Compared to social bees, solitary species show strong differences in their lifestyle and behavior, in particular regarding their nesting and offspring recruitment. For most species investigated in this study, such as *Osmia* and *Heriades* spp., eggs are laid onto pollen provisions within small cavities, mostly of dead wood materials, and each cell is individually closed by a mixture of resin, stones, loam, and/or plant fibers [8]. The material choice is distinct for different solitary bee species. Within each cell, only one egg is laid, but multiple cells may follow within the same cavity. This nesting procedure impairs nursing by the mother or sister bees, and larvae develop without further support after cell closure. This is a strong difference to social bees, where nurses actively take care of the offspring. Nursing also has importance in controlling microbial agents including pathogens [9]. A strategy of social bee pathogens, such as *Paenibacillus*, might thus be to maintain latent populations in solitary bees that share similar flower resources for pollen collection with social bees [10–12]. It has been shown that horizontal transmission of *P*. *larvae* spores is dependent on honey bee colony density and distance between hives [13], but wild and especially solitary bees as vectors have so far not been investigated.

*Paenibacillus* bacteria are also commonly found in the plant rhizosphere where they are well-known for their beneficial effects on the plant host. The benefit is primarily due to anti-microbial capacities and with that a strong factor in reducing risk of several plant diseases [14–16]. *Paenibacillus* bacteria are also capable of nutrient allocation (nitrogen fixation) and bioremediation [17–19]. Given the conditions of a solitary bee nest described above, the humid, enclosed, untended, and nutrient-rich environment may be an excellent growing ground for molds and other harmful microbes. So far, it has not been investigated, to our knowledge, whether the Paenibacilli found in association with wild bees and their nests belong to the pathogenic strains with latent virulent populations for honey bees or others with functional potential of avail.

We thus (1a) screened eight solitary bee nests of a cavity-breeding solitary bee species for occurrence of *Paenibacillus* with cultivation-independent, high-throughput sequencing-based 16S metabarcoding. We differentiated between nesting materials, pollen, and larvae to conclude whether the bacteria likely originate from flowers or nest material origins. (1b) We additionally screened 78 adult wild bee specimens from the same location, mostly solitaries, and distinguished gut and surface microbial communities for each. (2) We isolated a *Paenibacillus* strain with 100% 16S sequence identity for the full marker length of the 16S screening (V4) and sequenced its genome. We were interested in whether we can (2a) find virulence factors known from foulbrood causatives or (2b) genes involved in anti-microbial activity. Since (2b) was positive, we additionally performed in vitro bioassays to confirm the bioactivity and to determine the effect on gram-positive and gram-negative bacteria as well as fungi. (3) Lastly, we were interested in how this strain is related to known bee-virulent and plant-beneficial strains and performed a phylogenomic analysis together with all other publicly available genomes from the family Paenibacillaceae.

## Results

### 16S screening

**Fig. 1:**
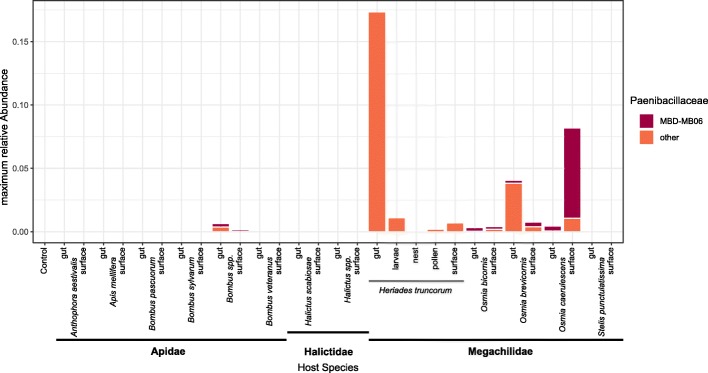
Occurrence of *Paenibacillus* MBD-MB06 within different bee species, separated by surface and gut tissues, and for *Heriades truncorum* additionally larvae, pollen, and nest materials. Values are maximum relative abundance of this bacterium with respect to the total microbiome assessment. Additionally, accumulative abundance of other Paenibacillaceae OTUs identified within the samples are stacked below

In total, we sequenced 202 samples from 13 bee species and six laboratory control samples consisting of the used extraction kit, PCRs, and laboratory water. We differentiated between adult surfaces (120), adult guts (62), larvae (8), pollen (4), and nest materials (8). Sequencing output was 4,431,246 cleaned-up reads after filtering and a mean of 22,001 per sample (± standard deviation 10,035). We could distinguish 20 *Paenibacillaceae* operational taxonomic units (OTUs), including *Paenibacillus*, *Cohnella*, *Aneurinibacillus*, and *Brevibacillus.* We did not, however, find any traces of such in the negative controls. The second most abundant *Paenibacillus* was with 100% sequence identity our isolate *Paenibacillus* MBD-MB06, which underwent genome sequencing. It was present with considerable abundances on surfaces of adult *Osmia caerulescens* (Fig. 1). It was, however, with lower abundances also found in nests, pollen, and guts and on the surface of various bee species, in particular other Megachilid bees (Fig. 1). Another *Paenibacillus* OTU also showed strong appearances especially in *Heriades truncorum* guts and larvae. All other *Paenibacillaceae* OTUs only occurred with marginal abundances. No OTU at all showed 100% sequence identity to any of the three detrimental strains.

### Genomics

The SPAdes assembly yielded 1251 scaffolds with a total length of 6,238,623 bp. After filtering 37 scaffolds with less than 1000 bp, a total length of 5,671,027 bp remained. The longest scaffold had a length of 1,316,040 bp while the scaffold N50 was 488,749 bp and N90 was 210,131 bp. The GC content of those sequences was 45.56%. The per base coverage of all remaining scaffolds (converted from k-mer coverage reported by SPAdes) was between 168× and 3500× with a median of 225×. The PROKKA pipeline annotated a total of 5103 genes (104 tRNAs, 1 tmRNA, 13 rRNAs, and 4985 CDSs). The 13 annotated rRNA genes correspond to 12 copies of 5S rRNA and one partial 16S sequence. No continuous 23S rRNA gene was annotated on the scaffolds, due to copy variation and filtering of reads below 1000 bp. The SPAdes assembly graph shows many branching events on nodes with similarity to rRNA genes (determined via BLAST in bandage, see Additional file 1). Coverage of the nodes suggests that there are roughly 15 copies of 16S and 23S rRNA genes. We manually selected representative sequences using the assembly graph. Those representative sequences follow the path with the highest coverage in the assembly graph, but there might be no single copy in the genome that has exactly this sequence. Therefore, we also chose to infer phylogeny using a whole genome approach rather than the 16S region alone.

### Phylogenomics

**Fig. 2:**
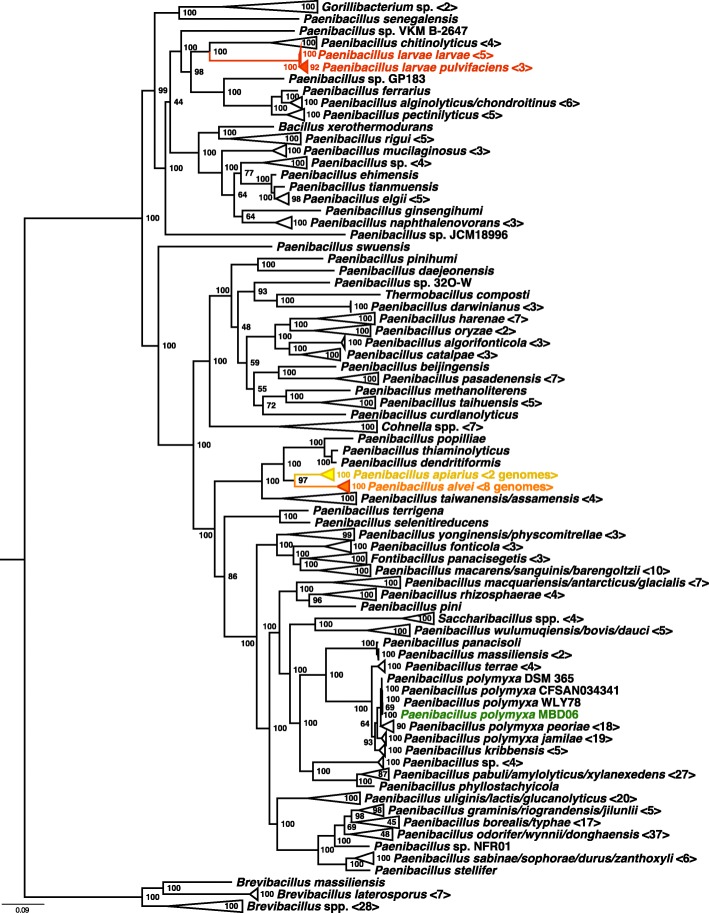
Phylogenomic tree of *Paenibacillus* MBD-MB06 and 367 other genomes of the Paenibacillaceae obtained with bcgTree [59]. Branches neither closely related to pathogeneous strains (red), secondary invaders (orange), suspected to be pathogeneous (yellow), nor our sequenced strain (green) were collapsed to ease facility of inspection. For collapses that included multiple species, those were separated by slashes. Numbers of genomes within the collapses are in brackets. An un-collapsed tree is provided as Additional file 6. Node values are maximum likelihood bootstrap support values (1000 replicates)

The final tree contained 367 taxa from the Paenibacillaceae plus our *Paenibacillus* MBD-MB06, including eight different genera, and was based on 107 core genes obtained from whole genomes (Fig. 2). Our strain clustered into the group of *Paenibacillus polymyxa*. Interestingly, the three pathogenic or secondarily invading species were not clustered all together, but split into two groups. The phylogenetic distance with respect to topology of the tree to the pathogenic *P*. *larvae* subsp. *larvae* and *P*. *larvae* subsp. *pulvifaciens* [20] strains was very high, and it showed a closer but still separated relationship to the *P*. *apiarius* and *P*. *alvei* strains. On the other hand, it seems to be closely related to plant-beneficial strains commonly found in the rhizosphere.

### Virulence factor screening

We found various genes related to general virulence and toxicity of bacteria towards insects, most of them however unrelated to specific bee diseases and widespread in Firmicutes: ESAT-6 secretion system extracellular protein A (EsxA, 85% identity), delta-endotoxin (cry-proteins, 2× ~ 40% identity) and enterotoxin (5× 25–40% identity). We further found homologies to protein domains that were named in the context of foulbrood diseases: a hypothetical protein with ricin-type lectin homology (29% identity) and enolase (68% and 87% identity), which are immunogenic and have been reported to be secreted proteins during pathogenesis of the American foulbrood. We further found homologies to chitin-binding proteins (5× 30–42% identity) considered non-catalytic, although we did not find any of the 22 screened chitinases. Beyond that, no other homologies to reported virulence factors of known bee disease-relevant *Paenibacillus* strains were identified.

### Genomic anti-microbial capabilities

The secondary metabolite analysis with antiSMASH predicted 27 gene clusters related to secondary metabolism. These were mostly “nonribosomal peptide synthetase cluster” (19) but also bacteriocin, lassopeptide, lantipeptide, type I PKS cluster, trans-AT PKS cluster, and “other.” Among the most similar known clusters were biosynthetic gene clusters for polymyxin, tridecaptin, fusaricidin, and oaenibacterin, compounds well-known for their anti-microbial effects.

### Anti-microbial bioassays

**Fig. 3:**
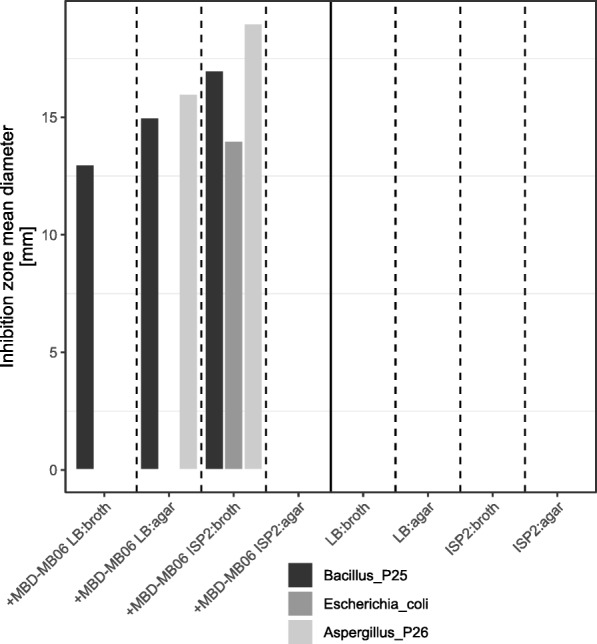
Anti-microbial activities of *Paenibacillus* MBD-MB06 measured by inhibition zone diameter [mm] (mean of *N* = 3 different plates and 2 different discs)

The ethyl acetate extract from LB broth inhibited only the growth of the tested gram-positive bacterium, and the extract from LB agar inhibited both the gram-positive bacteria and the fungus, while the crude extract from ISP2-agar inhibited all of the test organisms (Fig. 3). These results confirm the anti-microbial bioactivity of *Paenibacillus* MBD-MB06 as suggested by the genome.

## Discussion

We found *Paenibacillus* to be a common, but irregularly occurring member of wild bee microbiomes and their nesting materials, although mostly in low relative abundance with respect to the whole microbiome. It is thus probably not an obligatory part of the microbiome for the investigated species, but its presence may be dependent on several factors, including landscapes, co-occurring species, behavior, and visited flower microbiomes [10, 12, 21]. Interestingly, *Paenibacillus* was not found in high numbers in nest samples in our metabarcoding approach, although we were able to cultivate it from these materials. This suggests that they do not maintain a high density in comparison to the other diverse bacteria in the nests [5, 10], but are likely still present and active in them. Their origin is probably dependent on the environment, with nesting materials for some species originating from soils [5, 8] or shared floral resources [11, 12] with other insects that host these bacteria in stronger abundances.

One such bee is likely *Osmia caerulescens*, where we found our strain *Paenibacillus* MBD-MB06 consistently in considerable abundances on body surfaces. It has been demonstrated for different strains of *P*. *polymyxa* that they are capable of forming biofilms on host plant tissues [15], so the speculation here is that this might also be the case on the bee cuticula. Our finding of five genes encoding for chitin-binding proteins suggests the ability of the bacterium to bind to chitin and thus potentially to surface structures of insects. We currently have no experimental evidence for this specific strain, yet this has been demonstrated for other members of the genus, including *P*. *larvae* [22]. The lack of chitinases, i.e., chitin-degrading proteins, in our sequenced genome however suggests that there is no activity in degrading the body surface in contrast to genomes of strains inflicting this honey bee disease [22]. Using our phylogenomic approach, we were able to categorize the found *Paenibacillus* MBD-MB06 into a cluster of bacteria well-known to be able to produce such beneficial biofilms in plant-microbe interactions. These biofilms inhibit fungal colonization [15] and help against fungal penetration and finally infection [23]. This result opens new questions regarding the importance of cuticular microbiomes for insect health, which is currently mostly understudied because of the main research focus on insect gut microbiomes [24]. For other animals, many studies confirm that skin microbiomes generally also provide important functions to their hosts [25], which might also be the case for insects.

Some of the strains closely related to the one investigated here are reported to be able to produce secondary metabolites that show anti-microbial activity [14–16]. For example, the macrolide paenimacrolidin was isolated from *Paenibacillus* sp. F6-B70 and exhibited potent activity against methicillin-resistant *Staphylococcus aureus* [26]. Furthermore, we found in the sequenced genome the genetic capabilities to produce polymyxin, tridecaptin, fusaricidin, and paenibacterin, which all present potent anti-microbial compounds [27–29]. We were able to confirm such activity with in vitro assays and interestingly found that activity against gram-positive, gram-negative, and fungi differed according to changing cultivation media. Overall, the bacterium shows a diverse repertoire of natural products to control microbial agents that might be active depending on the specific environment. Given this, an interesting aspect for follow-up studies would be to measure transcription levels of relevant genes, not only the genomic presence, but also the actual concentration of these compounds within wild bees and their nests under natural conditions. Further, quantification studies on the strengths of repressions for these compounds against individual known bee pathogens, or whether the latter show resistances to such, might help to deepen our understanding about microbiome-mediated immunity in bees.

The phylogenomic analysis also confirmed a distinct separation from the harmful strains of *P. larvae*, *P*. *alvei*, and *P*. *apiarius* with a comparably distant phylogenetic relationship in the topology to each. Interestingly, these pathogenic or secondarily invading taxa clustered in two distinct groups, with *P. alvei* and *P. apiarius* clustering together and *P. larvae* being isolated in the tree from these. Regarding the topology of the whole tree, we found a further interesting result: the monophyly of the genus *Paenibacillus* has recently been questioned [30], and our phylogenomic tree supports the endeavor of taxonomic reassessment of this group by placing the genera *Cohnella*, *Thermobacillus*, *Gorillibacterium*, and *Saccharibacillus* with this genus [3].

We found few genes that encode for putative insecticidal toxins, some reported to co-occur with the American foulbrood disease (enolase, ricin-like lectin) [31, 32] and others unrelated yet still potentially harmful for insects in general (homologies to cry-proteins, ESAT-6 secretion system, and enterotoxins). We found non-catalytic chitin-binding proteins that might be supportive to chitin-degrading processes in insect pathogenesis [33], yet no evidence for chitinases themselves. The general affinity to chitin with a variety of chitin-binding genes, probably conserved throughout the genus given the homologs occurring in different parts of the tree, suggests a general ancestral tendency to associate with chitin-producing organisms, i.e., arthropods or fungi. Due to a lack of chitinases, as in our investigated strain, this might not necessarily be harmful, as known from the rhizosphere. The genome itself did not harbor any of the other key virulence factors (PlCBP49, C3larvin, SplA, toxB, PA14, Plx1-7, ETX, MTX2) reported for foulbrood agents [31, 32, 34]. The conserved potential within the genus to produce putative insecticidal toxins or precursors of such, which are also found as byproducts during foulbrood diseases, might however ease the evolution of pathogens within the genus and indicate some common genomic ancestry of disease-related genes.

*Paenibacillus* MBD-MB06 itself is unlikely to pose a latent threat to honey bees, even when sharing the same floral resources with its wild bee hosts [11, 12]. This does not exclude the fact that harmful pathogens may be vectorized through flowers and wild bees (indeed we found multiple functionally uncharacterized *Paenibacillus* bacteria present in our samples), or the risks of horizontal gene transfer under specific situations. It shows however that pathogenicity is not the default association between bees and the genus *Paenibacillus*. More likely, it is a generally neutral bacterium irregularly occurring as a non-obligate microbiome member in nests and on surfaces of a variety of wild bee species [5–7], and as a regular associate to particular species (here *Osmia caerulescens*). Due to its activity in the production of anti-microbial substances and genomic indications that it is able to bind chitin and produce biofilms as a barrier to fungal penetration, it might however, both in loose and tight associations, contribute to host health in certain situations, e.g., against mold infestations of nests and adults. We did not consider nutrient allocation or bioremediation capabilities in this study, which might additionally increase the benefit to host *Paenibacillus* for bees [17–19], particularly considering the diverse natural biochemistry of flowers and resins they are in contact with [35, 36], but also human-introduced insecticide, pesticide, and herbicide use in the environment threatening bee populations [37, 38]. Given the continuous process of adaptations between hosts and microbes [39] and shared genomic features of symbionts and pathogens [40], harmful and non-virulent *Paenibacillus* may both have a long co-evolutionary history with bees. Better understanding about the non-virulent microbiome members may thus also help to understand the evolutionary history of the diseases [41].

Our results also strengthen the concept of the holobiont theory, which suggests that eukaryotes are regarded optimally together with their microorganisms as an ecological unit instead of a separation of individuals [42]. In particular, our results support that this also includes irregular, variable, and non-core bacteria, which might be obtained from the local environment or are present only in specific lineages of hosts. It is thus important to consider variation due to host ecology, behavior, and biogeography as a factor within the holobiont principle [42]; otherwise, important functional and evolutionary processes and implications might be overlooked.

## Conclusions

Members of the genus *Paenibacillus* are common but irregularly occurring members of wild bee microbiomes, present on adult body surfaces, guts, and within nests particularly within the megachilid species. The wild bee-associated *Paenibacillus* strain investigated here genomically was phylogenetically and functionally distinct from harmful members relevant to honey bee colony diseases and is thus unlikely a latent threat, although it shared few conserved proteins putatively toxic to insects that might indicate ancestral predisposition for evolution of insect pathogens within the group. By contrast, our strain showed strong anti-microbial capabilities and shows genomic potential for chitin-binding biofilm-forming, making it a useful associate to avoid fungal penetration of the bee cuticula and a beneficial inhabitant of nests to repress fungal threats in humid and nutrient-rich environments of wild bee nests.

## Materials and methods

### Sample collection

Artificial nest sites were placed in the University of Würzburg Botanical Garden in Würzburg, Germany (49° 45′ 55.9″ N 9° 55′ 57.2″ E), in July 2014 and were checked for occupancy after 3 weeks. Nest sites were made of 50–100 reed canes (length 15–20 cm), which were put into plastic drainage tubes in dense bundles. Reed canes provided nest sites of different sizes so as to allow capture of different solitary bee species and different body sizes. New nests were identified by nest caps, lid-like structures built by females to protect filled nests. Only capped nests were taken to the laboratory. Species were in general identified by investigating the nesting materials. The artificial nesting sites attracted only one species, *Heriades truncorum.* For eight individual nests, pollen, larvae, and nest materials were treated separately and taken from different reed canes. No pollen was left in four samples; therefore, only four samples were processed for this material. Additionally, we collected adult bees from flowers during the same year from March to September every 3 weeks and close to the nests, by catching individuals with an autoclaved falcon tube and freezing them immediately. To avoid misidentification of hard-to-distinguish *Bombus terrestris*, *B. lucorum*, and *B. cryptarum*, we further treated these as the *Bombus* spp. complex. For adults, surface swabs were taken with sterile cotton buds trenched with autoclaved water for DNA extraction. For larger bees, guts were isolated by dissection under a binocular (120 surface samples, 62 guts). In total, we collected 202 samples from 13 morphologically distinguishable bee species and 6 extraction kit/water/PCR controls.

### 16S screening

DNA was isolated using the NucleoSpin Soil Kit (Macherey-Nagel) following the manufacturer’s instructions. For PCR amplification and sample-specific labeling, we followed a dual-indexing strategy [43], which uses the gene-specific primers 515f: 5′-GTGCCAGCMGCCGCGGTAA-3′ and 806r: 5′-GGACTACHVGGGTWTCTAAT-3′. Attached to these primers are pad, linker, index, and adapters for binding to the Illumina MiSeq platform; details on the primer design can be found in Kozich et al. [43]. The complete primer sequences were forward: 5′-AATGATACGGCGACCACCGAGATCTACAC XXXXXXXX TATGGTAATT GT GTGCCAGCMGCCGCGGTAA-3′ and reverse: 5′-AAGCAGAAGACGGCATACGAGAT XXXXXXXX AGTCAGTCAG CC GGACTACHVGGGTWTCTAAT-3′ [43] where XXXXXXXX denotes index sequences (sample-specific combinations of forward and reverse index sequences). For each sample, we performed three separate 10-μl amplification reactions to avoid PCR bias [44], each reaction containing [45] 5 μl 2× Phusion Master Mix (New England Biolabs, Ipswich, MA, USA), 0.33 μM each of the forward and reverse primers (sample-specific index combinations; purchased from Eurofins MWG Operon, Huntsville, AL, USA), 3.34-μl PCR grade water, and 1-μl DNA template. PCR conditions were initial denaturation step at 95 °C for 4 min, 30 cycles of denaturation at 95 °C for 40 s, annealing at 55 °C for 30 s, and elongation at 72 °C for 1 min, followed by a final extension step at 72 °C for 5 min.

After PCR, the triplicate reactions were re-combined and 5 μl taken to check for successful amplification on a 1.5% agarose gel. PCR products were cleaned up and equalized in DNA amount (25 ng) using the SequalPrep^TM^ Normalisation Plate Kit (Invitrogen GmbH, Carlsbad, CA, USA). After this normalization step, 5 μl of each sample was combined to a common pool, which was quality checked with a Bioanalyzer High Sensitivity DNA Chip (Agilent Technologies, Santa Clara, CA, USA) and quantified using dsDNA High Sensitivity Assay (Life Technologies, Carlsbad, CA, USA). The final library was diluted to 8 pM and spiked with 5% PhiX Control Kit v3 as recommended in the Illumina Sample Preparation Guide (Illumina Inc., San Diego, CA, USA). Sequencing was performed on the Illumina MiSeq using 2 × 250 cycles v2 chemistry (Illumina Inc., San Diego, CA, USA). Included in the sequencing run were six laboratory controls (two empty DNA isolation with 60-μl PCR grade water, four negative PCR controls with water instead of DNA template), which were processed in the exact same way as the samples. Sequencing data is available at the EBI-SRA repository, under the project accession number PRJEB27239.

Sequences were demultiplexed by examination of their MID barcode with the Illumina device’s own workbench software. We filtered data only to high-quality reads (>Q30, 99.9% accuracy), and no ambiguous characters were included in the following downstream analyses. Only sequences larger than 200 bp in length were used. Cleaning of reads, i.e., removal of bad quality reads and filtering of chimeric artifacts, was performed with USEARCH v8.1 [46]. The same tool was used to cluster sequences to OTUs (97% identity) and taxonomically classify representatives hierarchically up to genus level (90% bootstrap confidence threshold), making use of the RDP training set v16 (https://sourceforge.net/projects/rdp-classifier/). OTU-table, metadata, and classifications are in Additional file 2. Rarefaction curves flattened sufficiently, indicating that sequencing coverage was adequate given the diversity of the system (Additional file 3).

### Bacterial cultivation

Swabs from nests, pollen provisions, and larvae were additionally cultivated aerobically on Petri dishes supplemented with R2A medium (Roth GmbH) and fungicide (cycloheximide, Sigma-Aldrich, 30 μg/l). After incubation for 2–4 days at 37 °C, different colony-forming units were characterized based on appearance and distinct morphotypes and cultivated on separate LB agar plates containing no fungicide (LB medium and bacterial agar, AppliChem). For identification, DNA was extracted with the Fungal/Bacterial DNA MiniPrep kit (Zymo Research Corporation, Irvine CA, USA). A 16S rDNA gene PCR was run with 5 × 10 μl reactions of 5 μl Phusion Master Mix (5.0 μl; Biozym, Oldendorf, Germany), 0.25 μl of each primer, 3.5 μl bidest. H_2_O, and 1 μl of the eluted DNA. Used primers were forward 16S_27_f (5′-AGAGTTTGATCMTGGCTCAG-3′) and reverse 16S_1492_r (5′-TACGGYTACCTTGTTACGACTT-3′) (Eurofins Genomics, Ebersberg, Germany). PCR conditions contained an initial denaturation at 95 °C for 4 min, followed by 30 cycles of denaturation at 95 °C for 40 s, annealing at 53 °C for 30 s, and elongation at 72 °C for 1 min. Again, separate reactions were combined after PCR and 5 μl was used for quality control on a 1.5% agarose gel.

Samples were cleaned up with the NucleoMaq NGS Clean-up kit (Macherey-Nagel, Düren, Germany), and quantification was performed by means of the Qubit dsDNA assay and the Qubit Fluorometer (Life Technologies/Invitrogen, Carlsbad, CA, USA) to ensure that the needed concentration of 6 ng/μl is provided for DNA sequencing. 16S Sanger sequencing for strain identification was outsourced to StarSeq (Mainz, Germany).

Obtained sequences were identified using BLASTn [47] against GenBank [48] and compared to the metabarcoding data to obtain a strain with 100% identical hit over the complete sequence length with *Paenibacillus* from our 16S diversity assessments. We positively verified that no mismatches between the 16S of our isolated strain and the primers used for metabarcoding were present, to make sure that no bias against *Paenibacillus* was introduced. Furthermore, we checked that species of *Paenibacillus* are distinguishable by at least one single nucleotide polymorphism within the marker region (Additional file 4). The strain, hereafter referred to as *Paenibacillus* strain MBD-MB06, was then cultured in LB medium without fungicide, and a permanent Glycerol stock was stored at − 80 °C.

### Genome sequencing, assembly, annotation, and phylogenomics

High molecular weight DNA was cleaned up with the DNA Clean & Concentrator kit (Zymo Research). The genomic DNA library for the Illumina platform was generated using Nextera XT (Illumina Inc.) according to the manufacturer’s instructions. After tagmentation, size selection was performed using NucleoMag NGS Clean-up and Size Select (Macherey-Nagel) to obtain a library with median insert size around 500 bp. After PCR enrichment, the library was validated with a high-sensitivity DNA chip and Bioanalyzer 2100 (both Agilent Technologies, Inc.) and additionally quantified using the Qubit dsDNA HS assay (Life Technologies). It was sequenced on a MiSeq device using v2 2 × 250 bp chemistry, multiplexed together with five other bacterial genomes from different sources. Multiplexing was done via dual indexing, with the official Nextera indices N706 and S503.

Genomes were assembled by means of SPAdes (version 3.10.1) [49] using default settings on an 80-core Ubuntu 16.04 system with 250 GB of RAM. The resulting scaffolds were filtered by length (minimum 1000 bp) and coverage (minimum 10×) with SeqFilter (version 2.1.7) [50] after analysis with blobtools (version 1.0) [51]. Annotation was performed with the PROKKA pipeline (version 1.13) [52] including rRNA prediction with barrnap (version 0.8, https://github.com/tseemann/barrnap) and tRNA prediction with aragorn (version 1.2) [53]. Representative sequences for the 16S and 23S rRNA genes were manually extracted from the SPAdes assembly graph using bandage (version 0.8.0, Additional file 1) [54]. The genome was deposited publicly at the EBI-SRA with accession PRJEB27241.

Further, we performed an antiSMASH [55] analysis to identify bioactive compound production genes and related operons. Bee disease-related and general virulence factor genes were determined from literature [31, 32, 34] (PlCBP49, C3larvin, SplA, toxB, Ricin, PA14, Plx1-7, ETX, MTX2) and GenBank annotations (chitin-binding, chitinase, enhancin, enolase, toxin, virulence). Their amino acid sequences were obtained from genomes deposited at Genbank [48] (23 *P. alvei* accessions for 3 strains, 1 *P. apiarius* accession, 29 *P. larvae* subsp*. larvae* for 9 strains and 6 *P. larvae* subsp. *pulvifaciens* accessions for 3 strains, complete list in Additional file 5) and compared with our assembled genome using BLASTp [56] at an *e* value threshold of 0.0001. Positive hits were analyzed with SMART including PFAM domains to identify homologous domains [57].

Regarding the reconstruction of the phylogenetic position of our strain, we found multiple copies of 16S sequences within the genome, likewise to other members of the genus. Therefore, we performed a genome-based analysis of relationship to 367 other Paenibacillaceae (downloaded from ezbiocloud [58]) with the bacterial phylogenomics tool bcgTree [59] (maximum likelihood; 1000 bootstrap replications), rather than relying on the 16S marker.

### Anti-microbial bioassays

The anti-microbial activity was measured by the standard disc diffusion assay against gram-positive bacterium *Bacillus* sp. P25, gram-negative bacterium *Escherichia coli*, and the fungus *Aspergillus* sp. P26, all of which have been isolated from plants from the same botanical garden in a different study of URA. We aimed to optimize the production of the antibiotic metabolites by the “one-strain-many-compounds” (OSMAC) approach [60, 61] using different fermentation conditions. *Paenibacillus* MBD-MB06 was fermented in two different media, LB and ISP2, as well as in broth and solid approaches to look for the diversity of the bioactive compounds that are produced by various approaches. Negative controls without *Paenibacillus* MBD-MB06 of all these variants were added in parallel on the same petri dishes.

The strain was fermented in an Erlenmeyer flask (100 ml), containing 50 ml of medium and incubated at 30 °C for 1 day with shaking at 150 rpm. After fermentation, filtration was done using Whatman filter paper (0.2-μm pore size filter, A. Hartenstein, Würzburg, Germany), and the supernatant was extracted for 10 min using a separating funnel with ethyl acetate (2 × 100 ml) to receive an ethyl acetate extract. For the solid fermentation experiment, 20 agar plates with the medium (square 120 × 120 mm) were inoculated with 100 μl of overnight cultures of *Paenibacillus* MBD-MB06 and incubated at 30 °C for 1 day. The agar media with bacterial biomass were scalped into small pieces and transferred to a 500-ml Erlenmeyer flask. Two hundred milliliters of ethyl acetate/flask was added to submerge the agar pieces and macerate the medium culture under shaking at 150 rpm for overnight. The macerations were subsequently filtered by gravity using Whatman filter paper (0.2-μm pore size filter, A. Hartenstein, Germany). The filtrates were combined and evaporated under vacuum (Büchi, Germany) to give the ethyl acetate extracts. The organic extracts (20 mg/ml) were then tested against three pathogens in vitro. For this, sterile filter discs (6 mm) impregnated with the ethyl acetate extracts (25 μl, three times) were placed on agar plates that had been inoculated with the test pathogen. Adjusted inoculums of each microorganism, corresponding to 0.5 McFarland turbidity, were used. Ampicillin and gentamicin were used as the antibacterial standards (gram-positive and gram-negative, respectively), while methanol served as the negative control. After 24-h incubation at 37 °C (bacteria) and 30 °C (fungus), the anti-microbial potential was quantitatively assessed as diameter of the inhibition zone (each three different plates and two filter discs).

## Additional files

Additional file 1: Presence of multiple ribosomal genes within the genome of *Paenibacillus* MBD06. Coverage of the nodes suggests that there are roughly 15 copies of 16S and 23S rRNA genes. Due to dissimilarities of the copies, SPAdes did not return contigs with full length 16S or 23S. Instead, we selected representative sequences manually: Those representative sequences follow the path with highest coverage in the assembly graph, but there might be no single copy in the genome that has exactly this sequence. The supplement includes an extract of the visualized assembly graph, as well as the representative sequences. (DOCX 460 kb)

Additional file 2: OTU tables, metadata, and taxonomic classification. (ZIP 125 kb)

Additional file 3: Rarefaction analysis for each of the samples, showing sufficient flattening of new OTU detection to assess the diversity. (PDF 155 kb)

Additional file 4: We used 406 16S V4 sequences from the genus *Paenibacillus* (including one *Paenibacillus polymyxa*) from the ezbiocloud 16S rRNA database and 935 16S V4 sequences of *Paenibacillus polymyxa* strains from NCBI to assess the occurrences of interspecific and intraspecific single nucleotide polymorphisms. (DOCX 40 kb)

Additional file 5: List of GenBank accessions used for virulence screening, which include whole genome projects, partial genomes as well as scaffolds. (DOCX 14 kb)

Additional file 6: The same tree as Fig. 2, yet without collapsed branches. (PDF 24 kb)

## References

[1] Genersch E (2010). American Foulbrood in honeybees and its causative agent, *Paenibacillus* larvae. J Invertebr Pathol.

[2] Forsgren E (2010). European foulbrood in honey bees. J Invertebr Pathol.

[3] Grady EN, MacDonald J, Liu L, Richman A, Yuan Z-C. Current knowledge and perspectives of *Paenibacillus*: a review. Microb Cell Factories. 2016 cited 2018 Jun 7:15. 10.1186/s12934-016-0603-7 PMC5134293 27905924

[4] Katznelson H (1955). *Bacillus apiarius*, n. sp., an aerobic spore-forming organism isolated from honeybee larvae. J Bacteriol.

[5] Keller A, Grimmer G, Steffan-Dewenter I (2013). Diverse microbiota identified in whole intact nest chambers of the red mason bee *Osmia bicornis* (Linnaeus 1758). PLoS One.

[6] Voulgari-Kokota A, Grimmer G, Steffan-Dewenter I, Keller A (2019). Bacterial community structure and succession in nests of two megachilid bee genera. FEMS Microbiol Ecol.

[7] Lozo J, Berić T, Terzić-Vidojević A, Stanković S, Fira D, Stanisavljević L (2015). Microbiota associated with pollen, bee bread, larvae and adults of solitary bee *Osmia cornuta* (Hymenoptera: Megachilidae). Bull Entomol Res.

[8] Potts SG, Vulliamy B, Roberts S, O’Toole C, Dafni A, Ne’eman G (2005). Role of nesting resources in organising diverse bee communities in a Mediterranean landscape. Ecol Entomol.

[9] Gilliam M, Taber S, Lorenz BJ, Prest DB (1988). Factors affecting development of chalkbrood disease in colonies of honey bees, Apis mellifera, fed pollen contaminated with Ascosphaera apis. J Invertebr Pathol.

[10] Anderson KE, Sheehan TH, Mott BM, Maes P, Snyder L, Schwan MR (2013). Microbial ecology of the hive and pollination landscape: bacterial associates from floral nectar, the alimentary tract and stored food of honey bees (*Apis mellifera*). PLoS One.

[11] McFrederick QS, Thomas JM, Neff JL, Vuong HQ, Russell KA, Hale AR (2017). Flowers and wild megachilid bees share microbes. Microb Ecol.

[12] Junker RR, Keller A (2015). Microhabitat heterogeneity across leaves and flower organs promotes bacterial diversity. FEMS Microbiol Ecol.

[13] Lindström A, Korpela S, Fries I (2008). Horizontal transmission of *Paenibacillus larvae* spores between honey bee ( *Apis mellifera* ) colonies through robbing. Apidologie.

[14] Beatty PH, Jensen SE (2002). *Paenibacillus polymyxa* produces fusaricidin-type antifungal antibiotics active against Leptosphaeria maculans, the causative agent of blackleg disease of canola. Can J Microbiol.

[15] Haggag WM, Timmusk S (2008). Colonization of peanut roots by biofilm-forming *Paenibacillus polymyxa* initiates biocontrol against crown rot disease. J Appl Microbiol.

[16] Son SH, Khan Z, Kim SG, Kim YH (2009). Plant growth-promoting rhizobacteria, *Paenibacillus polymyxa* and *Paenibacillus lentimorbus* suppress disease complex caused by root-knot nematode and fusarium wilt fungus. J Appl Microbiol.

[17] Fu G, Li R, Wu X, Gao B, Yuan X, Wan C, et al. Glyphosate bioremediation of contaminated fish-pond water by *Paenibacillus sp.* FUJX 401 from industrial activated sludge. Paris: Atlantis Press; 2016. cited 2018 May 7

[18] Varjani SJ, Gnansounou E, Gurunathan B, Pant D, Zakaria ZA. Waste bioremediation. Singapore: Springer Verlag; 2017.

[19] Seldin L (2011). Paenibacillus, nitrogen fixation and soil fertility. Endospore-forming soil bacteria.

[20] Ashiralieva A, Genersch E (2006). Reclassification, genotypes and virulence of *Paenibacillus larvae*, the etiological agent of American foulbrood in honeybees - a review. Apidologie.

[21] Junker RR, Romeike T, Keller A, Langen D (2014). Density-dependent responses by bumblebees to flower dwelling bacteria. Apidologie.

[22] Garcia-Gonzalez E, Poppinga L, Fünfhaus A, Hertlein G, Hedtke K, Jakubowska A (2014). *Paenibacillus larvae* chitin-degrading protein PlCBP49 is a key virulence factor in American Foulbrood of honey bees. PLoS Pathog.

[23] Ortiz-Urquiza A, Keyhani NO (2013). Action on the surface: entomopathogenic fungi versus the insect cuticle. Insects.

[24] Engel P, Kwong WK, McFrederick Q, Anderson KE, Barribeau SM, Chandler JA (2016). The bee microbiome: impact on bee health and model for evolution and ecology of host-microbe interactions. MBio.

[25] Grice EA, Segre JA (2011). The skin microbiome. Nat Rev Microbiol.

[26] Wu X-C, Qian C-D, Fang H-H, Wen Y-P, Zhou J-Y, Zhan Z-J (2011). Paenimacrolidin, a novel macrolide antibiotic from *Paenibacillus sp.* F6-B70 active against methicillin-resistant *Staphylococcus aureus*. Microb Biotechnol.

[27] He Z, Kisla D, Zhang L, Yuan C, Green-Church KB, Yousef AE (2007). Isolation and identification of a *Paenibacillus polymyxa* strain that coproduces a novel lantibiotic and polymyxin. Appl Environ Microbiol.

[28] Choi S-K, Park S-Y, Kim R, Lee C-H, Kim JF, Park S-H (2008). Identification and functional analysis of the fusaricidin biosynthetic gene of *Paenibacillus polymyxa* E681. Biochem Biophys Res Commun.

[29] Lohans CT, Huang Z, van Belkum MJ, Giroud M, Sit CS, Steels EM (2012). Structural characterization of the highly cyclized lantibiotic paenicidin A via a partial desulfurization/reduction strategy. J Am Chem Soc.

[30] Zeigler DR, Perkins JB (2015). The genus Bacillus. Practical handbook of microbiology.

[31] Djukic M, Brzuszkiewicz E, Fünfhaus A, Voss J, Gollnow K, Poppinga L (2014). How to kill the honey bee larva: genomic potential and virulence mechanisms of *Paenibacillus larvae*. PLoS One.

[32] Antúnez K, Anido M, Arredondo D, Evans JD, Zunino P (2011). *Paenibacillus larvae* enolase as a virulence factor in honeybee larvae infection. Vet Microbiol.

[33] Frederiksen RF, Paspaliari DK, Larsen T, Storgaard BG, Larsen MH, Ingmer H (2013). Bacterial chitinases and chitin-binding proteins as virulence factors. Microbiology.

[34] Poppinga L, Genersch E. Molecular pathogenesis of American Foulbrood: how *Paenibacillus larvae* kills honey bee larvae. Curr Opin Insect Sci 2015;10:29–36. 10.1016/j.cois.2015.04.013 29588011

[35] Tiedeken EJ, Egan PA, Stevenson PC, Wright GA, Brown MJF, Power EF (2016). Nectar chemistry modulates the impact of an invasive plant on native pollinators. Funct Ecol.

[36] Kaluza BF, Wallace H, Keller A, Heard TA, Jeffers B, Drescher N (2017). Generalist social bees maximize diversity intake in plant species-rich and resource-abundant environments. Ecosphere.

[37] Gill RJ, Ramos-Rodriguez O, Raine NE (2012). Combined pesticide exposure severely affects individual- and colony-level traits in bees. Nature.

[38] Goulson D, Nicholls E, Botías C, Rotheray EL (2015). Bee declines driven by combined stress from parasites, pesticides, and lack of flowers. Science.

[39] Pérez-Brocal V, Latorre A, Symbionts MA (2011). Pathogens: what is the difference? Between pathogenicity and commensalism.

[40] Goebel W, Gross R (2001). Intracellular survival strategies of mutualistic and parasitic prokaryotes. Trends Microbiol.

[41] McClelland M, Sanderson KE, Clifton SW, Latreille P, Porwollik S, Sabo A (2004). Comparison of genome degradation in Paratyphi A and Typhi, human-restricted serovars of *Salmonella enterica* that cause typhoid. Nat Genet.

[42] Bordenstein SR, Theis KR (2015). Host biology in light of the microbiome: ten principles of holobionts and hologenomes. PLoS Biol.

[43] Kozich JJ, Westcott SL, Baxter NT, Highlander SK, Schloss PD (2013). Development of a dual-index sequencing strategy and curation pipeline for analyzing amplicon sequence data on the MiSeq Illumina sequencing platform. Appl Environ Microbiol.

[44] Sickel W, Ankenbrand M, Grimmer G, Holzschuh A, Härtel S, Lanzen J (2015). Increased efficiency in identifying mixed pollen samples by meta-barcoding with a dual-indexing approach. BMC Ecol.

[45] Sickel W, Steffan-Dewenter I, Meuche I, Grafe TU, Keller A (2016). Diet determines bacterial diversity and community structure in Bornean pitcher plants. Microb Ecol.

[46] Edgar RC (2010). Search and clustering orders of magnitude faster than BLAST. Bioinformatics.

[47] Altschul SF, Gish W, Miller W, Myers EW, Lipman DJ (1990). Basic local alignment search tool. J Mol Biol.

[48] Benson DA, Cavanaugh M, Clark K, Karsch-Mizrachi I, Lipman DJ, Ostell J (2013). GenBank. Nucleic Acids Res.

[49] Bankevich A, Nurk S, Antipov D, Gurevich AA, Dvorkin M, Kulikov AS (2012). SPAdes: a new genome assembly algorithm and its applications to single-cell sequencing. J Comput Biol.

[50] Hackl T, Hedrich R, Schultz J, Förster F (2014). proovread: large-scale high-accuracy PacBio correction through iterative short read consensus. Bioinformatics.

[51] Laetsch DR, Blaxter ML (2017). BlobTools: interrogation of genome assemblies. F1000Research.

[52] Seemann T (2014). Prokka: rapid prokaryotic genome annotation. Bioinformatics.

[53] Laslett D, Canback (2004). ARAGORN, a program to detect tRNA genes and tmRNA genes in nucleotide sequences. Nucleic Acids Res.

[54] Wick RR, Schultz MB, Zobel J, Holt KE (2015). Bandage: interactive visualization of de novo genome assemblies. Bioinformatics.

[55] Weber T, Blin K, Duddela S, Krug D, Kim HU, Bruccoleri R (2015). antiSMASH 3.0—a comprehensive resource for the genome mining of biosynthetic gene clusters. Nucleic Acids Res.

[56] Altschul SF, Madden TL, Schäffer AA, Zhang J, Zhang Z, Miller W (1997). Gapped BLAST and PSI-BLAST: a new generation of protein database search programs. Nucleic Acids Res.

[57] Schultz J, Milpetz F, Bork P, Ponting CP (1998). SMART, a simple modular architecture research tool: identification of signaling domains. PNAS.

[58] Yoon S-H, Ha S-M, Kwon S, Lim J, Kim Y, Seo H (2017). Introducing EzBioCloud: a taxonomically united database of 16S rRNA gene sequences and whole-genome assemblies. Int J Syst Evol Microbiol.

[59] Ankenbrand MJ, Keller A (2016). bcgTree: automatized phylogenetic tree building from bacterial core genomes. Genome.

[60] Abdelmohsen UR, Grkovic T, Balasubramanian S, Kamel MS, Quinn RJ, Hentschel U (2015). Elicitation of secondary metabolism in actinomycetes. Biotechnol Adv.

[61] Hemphill CFP, Sureechatchaiyan P, Kassack MU, Orfali RS, Lin W, Daletos G (2017). OSMAC approach leads to new fusarielin metabolites from *Fusarium tricinctum*. J Antibiot.

