# Supplementary material for: Wild bees and their nests host Paenibacillus bacteria with functional potential of avail
Source: Microbiome. 2018 Dec 22;6:229. doi: 10.1186/s40168-018-0614-1 (PMC6303958; doi:10.1186/s40168-018-0614-1)
Supplement: Supplementary file 1 — Presence of multiple ribosomal genes within the genome of Paenibacillus MBD06. Coverage of the nodes suggests that there are roughly 15 copies of 16S and 23S rRNA genes. Due to dissimilarities of the copies, SPAdes did not return contigs with full length 16S or 23S. Instead, we selected representative sequences manually: Those representative sequences follow the path with highest coverage in the assembly graph, but there might be no single copy in the genome that has exactly this sequence. The supplement includes an extract of the visualized assembly graph, as well as the representative sequences. (DOCX 460 kb) [file 40168_2018_614_MOESM1_ESM.docx]

*Additional file 1: Presence of multiple ribosomal genes within the genome of Paenibacillus MBD06. Coverage of the nodes suggests that there are roughly 15 copies of 16S and 23S rRNA genes. Due to dissimilarities of the copies, SPAdes did not return contigs with full length 16S or 23S. Instead we selected representative sequences manually: Those representative sequences follow the path with highest coverage in the assembly graph but there might be no single copy in the genome that has exactly this sequence.*

**
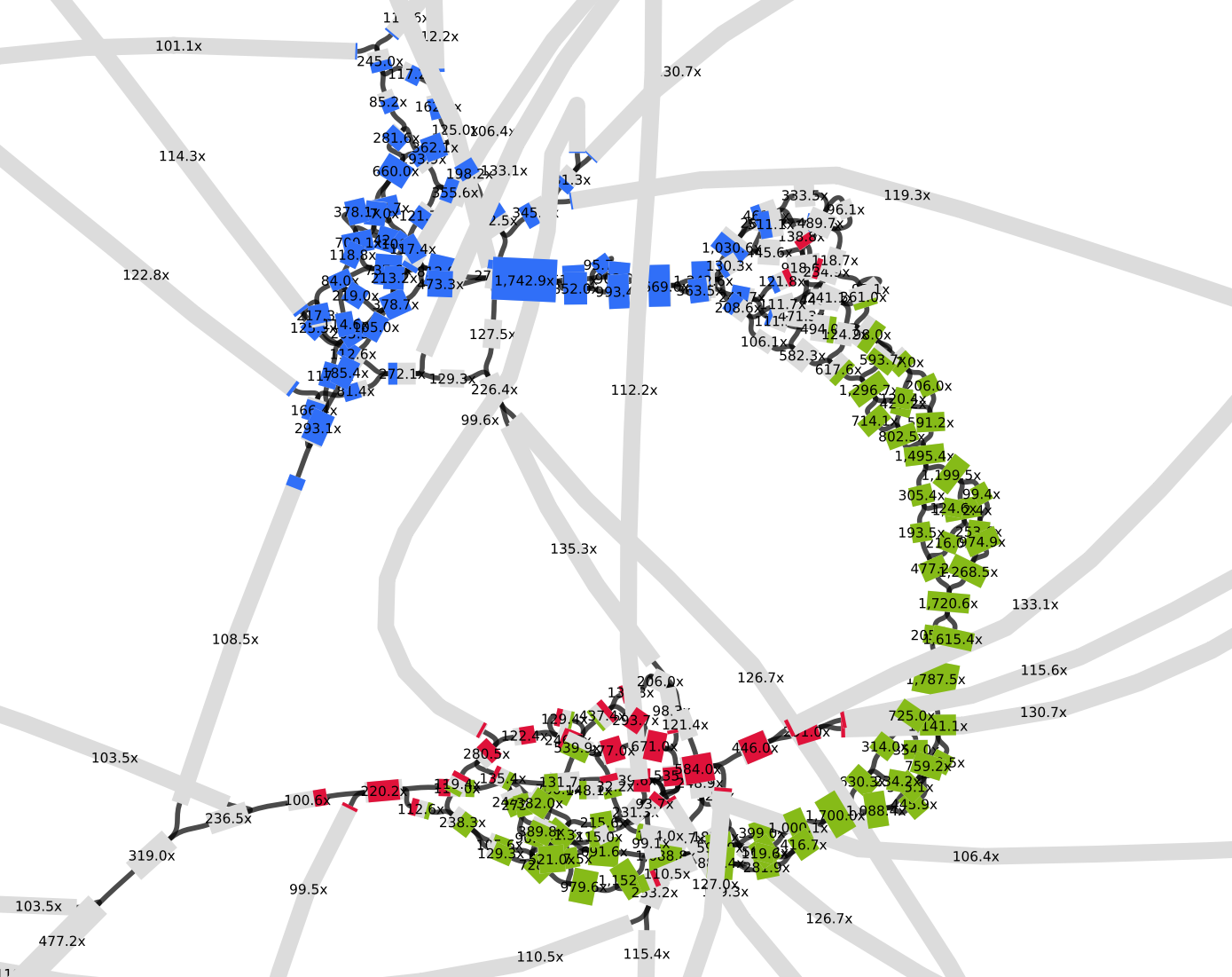
Figure S1:** Extract of the SPAdes assembly graph, visualized with bandage. Nodes are colored according to BLAST hits against rRNA genes of P. polymyxa (NC_014622.2, locus tags: PPSC2_RS28775 (16S), PPSC2_RS28780 (23S), PPSC2_RS28785 (5S)). green: 23S, blue: 16S, red: 5S, grey: no hit. Numbers on the nodes are the k-mer coverage reported by SPAdes.

# Representative 16S Sequence

ATCTCGTCAGTTTCAAAATGAGCTTATCGCTCTTTCTATAAACCAGCTTCGGTTGGTCTTTAATGGAGAGTTTGATCCTGGCTCAGGACGAACGCTGGCGGCGTGCCTAATACATGCAAGTCGAGCGGGGTTATTTAGAAGCTTGCTTCTAAATAACCTAGCGGCGGACGGGTGAGTAACACGTAGGCAACCTGCCCACAAGACAGGGATAACTACCGGAAACGGTAGCTAATACCCGATACATCCTTTTCCTGCATGGGCGAAGGAGGAAAGACGGAGCAATCTGTCACTTGTGGATGGGCCTGCGGCGCATTAGCTAGTTGGTGGGGTAAAGGCCTACCAAGGCGACGATGCGTAGCCGACCTGAGAGGGTGATCGGCCACACTGGGACTGAGACACGGCCCAGACTCCTACGGGAGGCAGCAGTAGGGAATCTTCCGCAATGGGCGAAAGCCTGACGGAGCAACGCCGCGTGAGTGATGAAGGTTTTCGGATCGTAAAGCTCTGTTGCCAGGGAAGAACGTCTTGTAGAGTAACTGCTACAAGAGTGACGGTACCTGAGAAGAAAGCCCCGGCTAACTACGTGCCAGCAGCCGCGGTAATACGTAGGGGGCAAGCGTTGTCCGGAATTATTGGGCGTAAAGCGCGCGCAGGCGGCTCTTTAAGTCTGGTGTTTAATCCCGAGGCTCAACTTCGGGTCGCACTGGAAACTGGGGAGCTTGAGTGCAGAAGAGGAGAGTGGAATTCCACGTGTAGCGGTGAAATGCGTAGAGATGTGGAGGAACACCAGTGGCGAAGGCGACTCTCTGGGCTGTAACTGACGCTGAGGCGCGAAAGCGTGGGGAGCAAACAGGATTAGATACCCTGGTAGTCCACGCCGTAAACGATGAATGCTAGGTGTTAGGGGTTTCGATACCCTTGGTGCCGAAGTTAACACATTAAGCATTCCGCCTGGGGAGTACGGTCGCAAGACTGAAACTCAAAGGAATTGACGGGGACCCGCACAAGCAGTGGAGTATGTGGTTTAATTCGAAGCAACGCGAAGAACCTTACCAGGTCTTGACATCCCTCTGACCGCTGTAGAGATATGGCTTTCCTTCGGGACAGAGGAGACAGGTGGTGCATGGTTGTCGTCAGCTCGTGTCGTGAGATGTTGGGTTAAGTCCCGCAACGAGCGCAACCCTTATGCTTAGTTGCCAGCAGGTCAAGCTGGGCACTCTAAGCAGACTGCCGGTGACAAACCGGAGGAAGGTGGGGATGACGTCAAATCATCATGCCCCTTATGACCTGGGCTACACACGTACTACAATGGCCGGTACAACGGGAAGCGAAGCCGCGAGGTGGAGCCAATCCTAGAAAAGCCGGTCTCAGTTCGGATTGTAGGCTGCAACTCGCCTACATGAAGTCGGAATTGCTAGTAATCGCGGATCAGCATGCCGCGGTGAATACGTTCCCGGGTCTTGTACACACCGCCCGTCACACCACGAGAGTTTACAACACCCGAAGTCGGTGGGGTAACCCGCAAGGGAGCCAGCCGCCGAAGGTGGGGTAGATGATTGGGGTGAAGTCGTAACAAGGTAGCCGTATCGGAAGGTGCGGCTGGATCACCTCCTTTCTATGGAGAATCGTTTCCTGCGATGGAAACATTCAAATTAGCAGGTACATGTACCTGCGACCGGATATTCAATTCGGTTCATCACATTCGTGTGAAT

# Representative 23S Sequence

TTTGGCAAAAATCATTCTTTATCGAACATCGACATTTTCTTTTATCAAAGAAAAGTCTAGGTTAAGCTACAAAGAGCACACGGAGGATGCCTAGGCGCCAGGAGCCGACGAAGGACGTGGCGAACAACGATAAGGCCTCGGGGAGCTGTAAGCAAGCTTTGATCCGGGGATGTCCGAATGGGGAAACCCGGCTGTCTTCATCGACAGTCACTACTCACTGAATTCATAGGTGAGTGAGAGGCAGACCAGGGGAACTGAAACATCTAAGTACCCTGAGGAAGAGAAAACAATAGTGATTCCGTCAGTAGCGGCGAGCGAACGCGGATTAGCCCAAACCAAGGAGCTTGCTCCTTGGGGTTGTGGGACGTCTCACATGGAGTTACAAAGGAACCGGTTAGATGAAGAGGTCTGGAAAGGCCCGCCAGAGAAGGTAAAAGCCCTGTAGTTCAAAACTTGTTCTCTCCGAGACGGATCCCGAGTAGTGCGGGGCACGTGAAACCCCGTATGAATCCGGCAGGACCATCTGCCAAGGCTAAATACTCCCTGGCGACCGATAGTGAAGCAGTACCGTGAGGGAAAGGTGAAAAGCACCCCGGAAGGGGAGTGAAATAGATCCTGAAACCGTGTGCTTACAAGAAGTCAGAGCCCTATTTATGGGTGATGGCGTGCCTTTTGTAGAATGAACCGGCGAGTTACGTTCCCGTGCAAGGTTAAGGTGAAGAGCTGAAGCCGCAGCGAAAGCGAGTCTGAATAGGGCGAATGAGTACGTGGACGTAGACCCGAAACCGGGTGATCTACCCCTGTCCAGGGTGAAGGTGCGGTAACACGCACTGGAGGCCCGAACCCACGCATGTTGAAAAATGCGGGGATGAGGTGGGGGTAGCGGAGAAATTCCAATCGAACCCGGAGATAGCTGGTTCTCCCCGAAATAGCTTTAGGGCTAGCCTCGGAAAGAAGAATCGTGGAGGTAGAGCACTGATTGGGTGCGGGGCCCGCAAGGGTTACCAAGCTCAGTCAAACTCCGAATGCCATAGATTTAGTTCCGGGAGTCAGACAGTGAGTGCTAAGATCCATTGTCGAAAGGGAAACAGCCCAGACCATCAGCTAAGGTCCCCAAGTGTGTGTTAAGTGGGAAAGGATGTGGAGTTGCACAGACAACCAGGATGTTGGCTTAGAAGCAGCCACCATTGAAAGAGTGCGTAATAGCTCACTGGTCGAGTGACTCTGCGCCGAAAATGTAACGGGGCTAAACACACCACCGAAGCTATGGCTTGATGCTTGCATCAGGGGTAGGGGAGCGTTGAATGCGGGTTGAAGGTGTACCGTAAGGAGCGCTGGACTGCATTCAAGTGAGAATGCCGGTATGAGTAACGAAAAGATCTGTGAGAATCAGATCCGCCGAAAGCCTAAGGGTTCCTGAGGAAGGTTCGTCCGCTCAGGGTAAGTCGGGACCTAAGGCGAGGCCGATAGGCGTAGTCGAAGGACAACAGGTCGAAATTCCTGTACCACCGTAATCCGTTATGAGCGATGGGGTGACGCAGTAGGGTAGTGACGCGGACGGATGGATGTCCGTCTAAGCAGTGAGGCTGGTGTGTAGGCAAATCCGCACATCGTTAAGGCTGGGCTGTGATGGGGAGCGAAAATTGTAGTAGCGAAGGTCATGATCTCAGACTGCCAAGAAAAGCCTCTAGCCAGGAGAAGGTGCCCGTACCGCAAACCGACACAGGTAGGCGAGAAGAGAATTCTAAGGCGCGCGGAAGAACTCTCGTTAAGGAACTCGGCAAAATGACCCCGTAACTTCGGGAGAAGGGGTGCCTCGGTAGGGTGAATAGCCCGAGGGGGCCGCAGTGAAAAGGCCCAAGCGACTGTTTAGCAAAAACACAGGTCTGTGCGAAGCCGCAAGGCGAAGTATACGGGCTGACGCCTGCCCGGTGCTGGAAGGTTAAGGGGAGTGGTAAGCCTTCGGGCGAAGCTATGAACCGAAGCCCCAGTAAACGGCGGCCGTAACTATAACGGTCCTAAGGTAGCGAAATTCCTTGTCAGGTAAATTCTGACCCGCACGAATGGCGTAACGACTTGGGCGCTGTCTCAACGAGAGATCCGGTGAAATTTTAATACCTGTGAAGATGCAGGTTACCCGCGACAAGACGGAAAGACCCCATGGAGCTTTACTGCAGCTTGATATTGAATTTGGGTACGATCTGTACAGGATAGGTGGGAGCCGTCGAACTTTGAGCGCCAGCTTGAAGGGAGGCATCCTTGGGATACCACCCTGATCGTATCTAGGTTCTAACTTGGTACCGTAAACCGGTGCGAGGACAGTGTCAGGTGGGCAGTTTGACTGGGGCGGTCGCCTCCTAAAGAGTAACGGAGGCGCCCCAAGGTTCCCTCAGAATGGTTGGAAATCATTCGAAGAGTGCAAAGGCAGAAGGGAGCTTGACTGCGAGACCTACAAGTCGAGCAGGGACGAAAGTCGGGCTTAGTGATCCGGTGGTACCGCATGGAAGGGCCATCGCTCAACGGATAAAAGCTACCCTGGGGATAACAGGCTTATCTCCCCCAAGAGTCCACATCGACGGGGAGGTTTGGCACCTCGATGTCGGCTCATCGCATCCTGGGGCTGAAGTAGGTCCCAAGGGTTGGGCTGTTCGCCCATTAAAGCGGTACGCGAGCTGGGTTCAGAACGTCGTGAGACAGTTCGGTCCCTATCTGTCGTGGGCGTAGGAAATTTGAGAGGAGCTGTCCTTAGTACGAGAGGACCGGGATGGACGTACCGCTGGTGTACCAGTTGTTCCGCCAGGAGCACCGCTGGGTAGCTATGTACGGAAGGGATAAGCGCTGAAAGCATCTAAGCGTGAAGCCCCCCTCAAGATGAGATTTCCCAGTATGTAAGACCCCTTGAAGACGACGAGGTAGATAGGTTGGGGGTGGAAGTGCAGTAATGCATGGAGCTGACCAATACTAATCGGTCGAGGGCTTATCCTAAGATAAAACGCAATGAGTTTCGGATCCAGTTTTCAGGGTGTAACCTTGAACGTATGTACAAGTGACATACAACAA
