# Supplementary figures and images for: Wild bees and their nests host Paenibacillus bacteria with functional potential of avail
Source: Microbiome. 2018 Dec 22;6:229. doi: 10.1186/s40168-018-0614-1 (PMC6303958; doi:10.1186/s40168-018-0614-1)

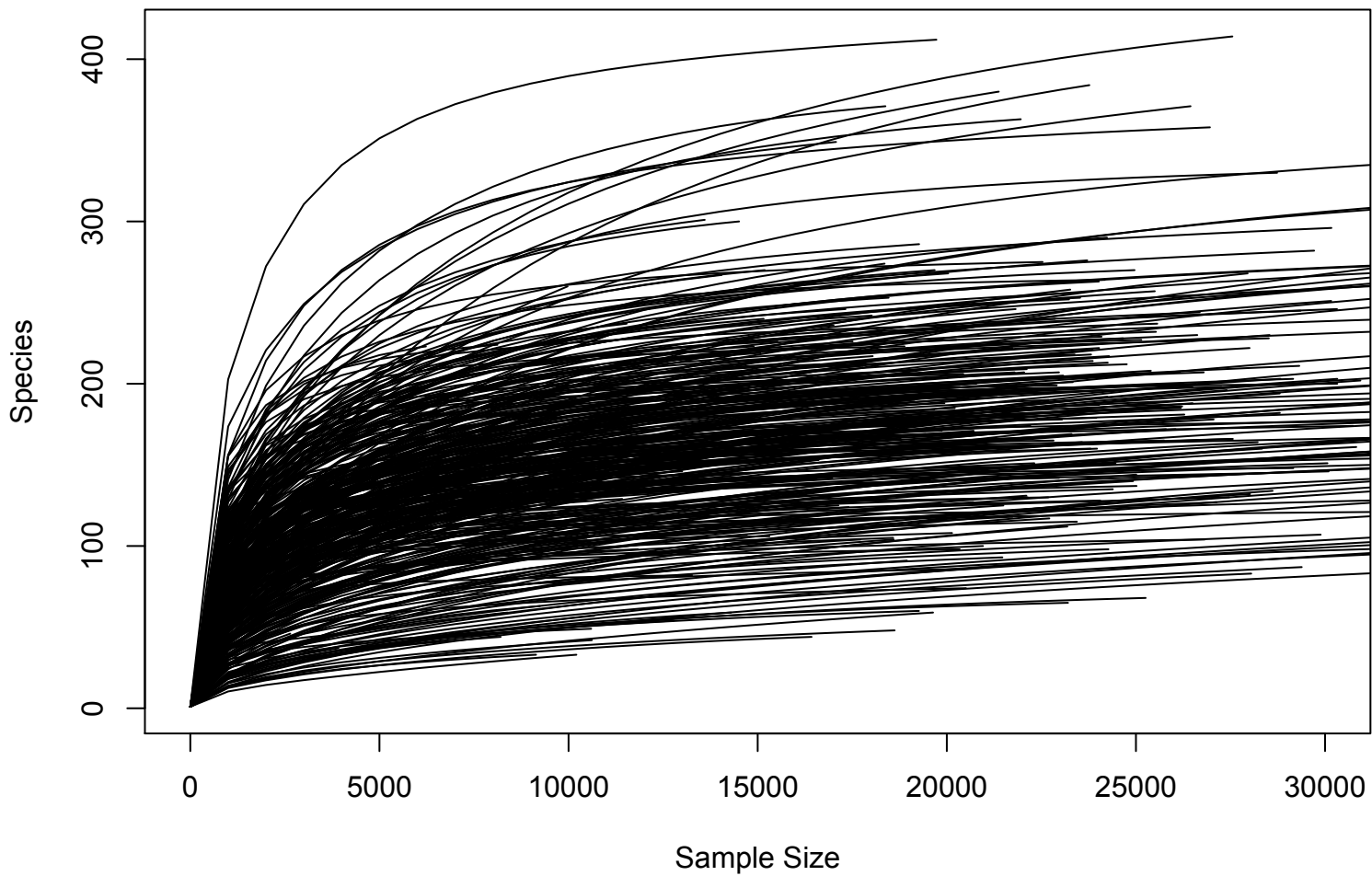

Supplement: Supplementary file 3 — Rarefaction analysis for each of the samples, showing sufficient flattening of new OTU detection to assess the diversity. (PDF 155 kb) [file 40168_2018_614_MOESM3_ESM.pdf]
