# Supplementary material for: Wild bees and their nests host Paenibacillus bacteria with functional potential of avail
Source: Microbiome. 2018 Dec 22;6:229. doi: 10.1186/s40168-018-0614-1 (PMC6303958; doi:10.1186/s40168-018-0614-1)
Supplement: Supplementary file 4 — We used 406 16S V4 sequences from the genus Paenibacillus (including one Paenibacillus polymyxa) from the ezbiocloud 16S rRNA database and 935 16S V4 sequences of Paenibacillus polymyxa strains from NCBI to assess the occurrences of interspecific and intraspecific single nucleotide polymorphisms. (DOCX 40 kb) [file 40168_2018_614_MOESM4_ESM.docx]

***Figure S3:*** *We used 406 16S V4 sequences from the genus Paenibacillus (including one Paenibacillus polymyxa) from the ezbiocloud 16S rRNA database (*[*https://www.ezbiocloud.net/resources/16s_download*](https://www.ezbiocloud.net/resources/16s_download)*, Updated: 2018.05) and 935 16S V4 sequences of Paenibacillus polymyxa strains from NCBI (*[*https://www.ncbi.nlm.nih.gov/nuccore*](https://www.ncbi.nlm.nih.gov/nuccore%5D%5D)*, on 2018-09-11) to assess the interspecific and intraspecific SNP rates compared to our variant. The sequences on NCBI have been searched with this term: "((Paenibacillus polymyxa[Organism]) AND 16S)". The V4 region has been extracted with dispr (*[*https://github.com/douglasgscofield/dispr*](https://github.com/douglasgscofield/dispr)*, commit 32670de, parameters: --pf V4:F:GTGCCAGCMGCCGCGGTAA --pr V4:R:GGACTACHVGGGTWTCTAAT --min 270 --max 310) Alignments were calculated with muscle (version v3.8.1551) and distance matrices computed with distmat (EMBOSS package version 6.6.0.0). Data preparation and plotting were performed in R (version 3.4.3) with ggplot2 (version 2.2.1).*

**References not in main text:**

Edgar, R.C. Muscle. Nucleic Acids Res 32(5), 1792-97

Rice, P. Longden, I. and Bleasby, A. EMBOSS. Trends in Genetics 16, (6) pp276--277

Wickham, H. ggplot2: Elegant Graphics for Data Analysis. Springer-Verlag New York, 2009
