# Supplementary material for: Wild bees and their nests host Paenibacillus bacteria with functional potential of avail
Source: Microbiome. 2018 Dec 22;6:229. doi: 10.1186/s40168-018-0614-1 (PMC6303958; doi:10.1186/s40168-018-0614-1)
Supplement: Supplementary file 5 — List of GenBank accessions used for virulence screening, which include whole genome projects, partial genomes as well as scaffolds. (DOCX 14 kb) [file 40168_2018_614_MOESM5_ESM.docx]

***Table S4:*** *List of GenBank accessions used for virulence screening, which include whole genome projects, partial genomes as well as scaffolds.*

**Paenibacillus alvei**

Paenibacillus alvei TS-15 PAALTS15_2, whole genome shotgun sequence. ATMT01000044

Paenibacillus alvei TS-15 PAALTS15_10, whole genome shotgun sequence. ATMT01000053

Paenibacillus alvei TS-15 PAALTS15_6, whole genome shotgun sequence. ATMT01000060

Paenibacillus alvei TS-15 PAALTS15_5, whole genome shotgun sequence. ATMT01000100

Paenibacillus alvei TS-15 PAALTS15_20, whole genome shotgun sequence. ATMT01000002

Paenibacillus alvei TS-15 PAALTS15_47, whole genome shotgun sequence. ATMT01000043

Paenibacillus alvei TS-15 PAALTS15_32, whole genome shotgun sequence. ATMT01000047

Paenibacillus alvei TS-15 PAALTS15_34, whole genome shotgun sequence. ATMT01000062

Paenibacillus alvei TS-15 PAALTS15_59, whole genome shotgun sequence. ATMT01000096

Paenibacillus alvei A6-6i-x PAAL66ix_1, whole genome shotgun sequence. ATMS01000085

Paenibacillus alvei A6-6i-x PAAL66ix_19, whole genome shotgun sequence. ATMS01000037

Paenibacillus alvei A6-6i-x PAAL66ix_2, whole genome shotgun sequence. ATMS01000086

Paenibacillus alvei A6-6i-x PAAL66ix_24, whole genome shotgun sequence. ATMS01000017

Paenibacillus alvei A6-6i-x PAAL66ix_6, whole genome shotgun sequence. ATMS01000026

Paenibacillus alvei A6-6i-x PAAL66ix_13, whole genome shotgun sequence. ATMS01000028

Paenibacillus alvei DSM 29 PAV_1c, whole genome shotgun sequence. AMBZ01000001

Paenibacillus alvei DSM 29 PAV_2c, whole genome shotgun sequence. AMBZ01000002

Paenibacillus alvei DSM 29 PAV_4c, whole genome shotgun sequence. NZ_AMBZ01000004

Paenibacillus alvei DSM 29 PAV_1c, whole genome shotgun sequence. NZ_AMBZ01000001

Paenibacillus alvei DSM 29 PAV_3c, whole genome shotgun sequence. AMBZ01000003

Paenibacillus alvei DSM 29 PAV_4c, whole genome shotgun sequence. AMBZ01000004

Paenibacillus alvei DSM 29 PAV_8c, whole genome shotgun sequence. AMBZ01000008

Paenibacillus alvei DSM 29 PAV_7c, whole genome shotgun sequence. AMBZ01000007

**Paenibacillus apiarius**

Paenibacillus apiarius strain NRRL B-23460 Ga0138518_103, whole genome shotgun sequence. NZ_NDGJ01000003

**Paenibacillus larvae subsp. larvae**

Paenibacillus larvae strain ATCC 9545 chitin-binding protein 49(CBP49) gene, complete cds. JX185746

Paenibacillus larvae subsp. larvae strain ATCC 9545 chromosome,complete genome. CP019687

Paenibacillus larvae subsp. larvae strain ATCC 9545 chromosome,complete genome. NZ_CP019687

Paenibacillus larvae strain DSM 25430 chitin-binding protein 49(CBP49) gene, complete cds. JX185745

Paenibacillus larvae subsp. larvae DSM 25430, complete genome. CP003355

Paenibacillus larvae subsp. larvae DSM 25430, complete genome. NC_023134

Paenibacillus larvae subsp. larvae DSM 25430 plasmid pPLA2_10, complete sequence. NC_023147

Paenibacillus larvae subsp. larvae DSM 25430 plasmid pPLA2_10, complete sequence. CP003356

Paenibacillus larvae subsp. larvae DSM 25430 5S ribosomal RNA, complete sequence. NR_121815

Paenibacillus larvae subsp. larvae DSM 25430 chromosome, complete genome. NZ_CP019652

Paenibacillus larvae subsp. larvae strain DSM 25430 23S ribosomalRNA gene, complete sequence. NR_121930

Paenibacillus larvae subsp. larvae DSM 25719 ERIC1_1c, whole genome shotgun sequence. ADFW01000001

Paenibacillus larvae subsp. larvae DSM 25719 ERIC1_1c, whole genome shotgun sequence. NZ_ADFW01000001

Paenibacillus larvae subsp. larvae DSM 25719 ERIC1_2c, whole genome shotgun sequence. ADFW01000002

Paenibacillus larvae subsp. larvae DSM 25719, whole genome shotgunsequencing project. NZ_ADFW00000000

Paenibacillus larvae subsp. larvae DSM 25719 plasmid pPLA1_10, whole genome shotgun sequence. NZ_ADFW01000008

Paenibacillus larvae subsp. larvae DSM 25719 ERIC1_7c, whole genome shotgun sequence. NZ_ADFW01000007 Paenibacillus larvae subsp. larvae DSM 25719 ERIC1_6c, whole genome shotgun sequence. NZ_ADFW01000006

Paenibacillus larvae subsp. larvae DSM 25719 ERIC1_5c, whole genome shotgun sequence. NZ_ADFW01000005

Paenibacillus larvae subsp. larvae DSM 25719 ERIC1_4c, whole genome shotgun sequence. NZ_ADFW01000004

Paenibacillus larvae subsp. larvae DSM 25719 ERIC1_3c, whole genome shotgun sequence. NZ_ADFW01000003

Paenibacillus larvae subsp. larvae DSM 25719 ERIC1_2c, whole genome shotgun sequence. NZ_ADFW01000002

Paenibacillus larvae subsp. larvae DSM 25719, whole genome shotgunsequencing project. ADFW00000000

Paenibacillus larvae subsp. larvae DSM 25719 ERIC1_3c, whole genome shotgun sequence. ADFW01000003

Paenibacillus larvae subsp. larvae DSM 25719 ERIC1_4c, whole genome shotgun sequence. ADFW01000004

Paenibacillus larvae subsp. larvae DSM 25719 ERIC1_5c, whole genome shotgun sequence. ADFW01000005

Paenibacillus larvae subsp. larvae DSM 25719 ERIC1_6c, whole genome shotgun sequence. ADFW01000006

Paenibacillus larvae subsp. larvae DSM 25719 ERIC1_7c, whole genome shotgun sequence. ADFW01000007

Paenibacillus larvae subsp. larvae DSM 25719 plasmid pPLA1_10, whole genome shotgun sequence. ADFW01000008

Paenibacillus larvae strain MEX14 contig00051, whole genome shotgun sequence. NZ_LAWY01000051

Paenibacillus larvae strain MEX14 contig00031, whole genome shotgun sequence. NZ_LAWY01000031

Paenibacillus larvae subsp. larvae strain Eric_IV chromosome,complete genome. NZ_CP019659

Paenibacillus larvae subsp. larvae strain Eric_IV chromosome,complete genome. CP019659

Paenibacillus larvae subsp. larvae strain Eric_III chromosome, complete genome. NZ_CP019655

Paenibacillus larvae subsp. larvae strain Eric_III chromosome, complete genome. CP019655

Paenibacillus larvae subsp. larvae strain ERIC_I chromosome, complete genome. NZ_CP019651

Paenibacillus larvae subsp. larvae strain ERIC_I chromosome, complete genome. CP019651

Paenibacillus larvae subsp. larvae B-3650 Seq193, whole genome shotgun sequence. NZ_ADZY03000230

Paenibacillus larvae subsp. larvae B-3650 Seq193, whole genome shotgun sequence. ADZY03000230

Paenibacillus larvae subsp. larvae B-3650 Contig368, whole genome shotgun sequence. ADZY03000002

Paenibacillus larvae subsp. larvae B-3650 Seq264, whole genome shotgun sequence. ADZY03000110

Paenibacillus larvae subsp. larvae B-3650 Contig375, whole genome shotgun sequence. ADZY03000155

Paenibacillus larvae subsp. larvae B-3650 Seq152, whole genome shotgun sequence. ADZY03000186

Paenibacillus larvae subsp. larvae B-3650 Seq348, whole genome shotgun sequence. ADZY03000192

Paenibacillus larvae subsp. larvae B-3650 Seq20, whole genome shotgun sequence. ADZY03000349

Paenibacillus larvae subsp. larvae BRL-230010 SCAFFOLD108, whole genome shotgun sequence. NZ_CH981479

Paenibacillus larvae subsp. larvae BRL-230010 SCAFFOLD62, whole genome shotgun sequence. NZ_CH981433

**Paenibacillus larvae subsp. pulvifaciens**

Paenibacillus larvae subsp. pulvifaciens strain CCM 38, complete genome. CP020327

Paenibacillus larvae subsp. pulvifaciens strain CCM 38, complete genome. NZ_CP020327

Paenibacillus larvae subsp. pulvifaciens strain SAG 10367chromosome, complete genome. NZ_CP020557

Paenibacillus larvae subsp. pulvifaciens strain SAG 10367chromosome, complete genome. CP020557

Paenibacillus larvae subsp. pulvifaciens strain ATCC 13537, complete genome. CP019794

Paenibacillus larvae subsp. pulvifaciens strain ATCC 13537, complete genome. NZ_CP019794
